# Drivers of Consumer Preference Derived from Active Volatiles for Cooked *Eriocheir sinensis*

**DOI:** 10.3390/ani13030541

**Published:** 2023-02-03

**Authors:** Qi Lu, Wei Ding, Xueqian Guo, Tong Xiao, Xichang Wang

**Affiliations:** 1 College of Food Science and Technology, Shanghai Ocean University, Shanghai 201306, China; 2 Shanghai Engineering Research Center of Aquaculture, Shanghai Ocean University, Shanghai 201306, China; 3 Shanghai Engineering Research Center of Aquatic-Product Processing and Preservation, Shanghai 201306, China

**Keywords:** *Eriocheir sinensis*, consumer acceptance, stepwise regression, just-about-right

## Abstract

**Simple Summary:**

Chinese mitten crab (*Eriocheir sinensis*) of different varieties and origins, is a popular aquatic product in Asian countries as a result of its unique flavor. Additionally, different genders and edible parts produce diverse volatile compounds that affect consumers’ choices and preferences. Therefore, it is essential to evaluate these differences to determine the factors impacting consumer preferences. This study explored the influence of exogenous and endogenous factors on volatile compounds and the sensory quality of farmed crabs from different rearing conditions and varieties. The volatile compounds differed among different origins and significantly influenced consumer preferences.

**Abstract:**

Consumers’ perception of volatiles determines their preferences and choices of food. Furthermore, various factors, such as varieties, origin, gender, and edible parts, may influence volatiles. The perception of edible parts of *E. sinensis* in two origins (Chongming and Taixing) regarding overall hedonic score (9-points), just-about-right scale (5-points), and intensity of attributes (7-points) were analyzed. The results showed that consumers preferred the hepatopancreas odor of female crabs in Chongming, with an overall preference of 6.84 and R_MAT_ (calculate the R-index by matrix) values >52.93%. The crabs’ origin (different feeding and rearing condition) was the primary reason for the odor profiles. The appropriate meaty and toasted odor in the hepatopancreas increased the overall preference by approximately 14.65–20.60%. Furthermore, 2-acetylthiazole, a volatile compound with a fatty odor, may significantly affect preferences and consumption behavior.

## 1. Introduction

With the increase in consumer awareness, consumers have been paying more attention to the sensory quality of food. The sensory quality benefits both consumers (empowers choice) and producers (increases consumer loyalty) [1]. The senses include color, odor, taste, and shape, with odor perception directly affecting consumers’ emotions. Notably, consumers’ first impression determines their preferences and brand choices [2]. Therefore, understanding odor attributes will facilitate developing targeted quality to satisfy consumer sensory demands and benefit producers, ultimately improving economic efficiency.

The Chinese mitten crab (*Eriocheir sinensis*) is an economically important aquatic product in Asian countries owing to its intense flavor and high nutritional value [3]. In China, crab culture yielded 808,274 tons of crab in 2021 (China Fishery Statistical Yearbook, 2022). Additionally, the diversity of varieties and rearing conditions have led to the establishment of numerous brands of *E. sinensis* [4]. Currently, five varieties of *E. sinensis* (Changjiang 1, Guanghe No. 1, Changjiang 2, Sea & River 21, and Noya No. 1) have been approved and promoted in China [5]. Furthermore, the coastal cultivation origins are mainly in the middle-lower Yangtze Plain, such as Shanghai, Anhui, Jiangsu, and Hubei. Notably, wild-caught crabs from the Yangtze River—a preferred brand among consumers—have long been the most expensive brand of *E. sinensis* [6]. The odor of aquatic products can be natural and/or derived from processing. The aquatic products carry a fishy and earthy odor, and the volatiles derived from the processing are of greater interest.

The flavor of *E. sinensis* has been extensively investigated and screened for 21 characteristic aromatic substances (aldehydes and ketones) in the gonads and hepatopancreas using principal component and correspondence analysis [7]. The volatile compound composition of the edible parts of *E. sinensis* with various feeding modes differed significantly and can be a distinguishing characteristic [8]. Specifically, 3-methyl-butanal, hexanal, nonanal, benzaldehyde, trimethylamine, naphthalene, 2-ethylfuran, 2-methylnaphthalene, and 1-methylnaphthalene were the primary contributors to the aroma profile of *E. sinensis* [9]. Currently, few studies have investigated the relationship between the flavor and consumer acceptance of *E. sinensis*. However, consumer acceptance studies may provide targeted directions for the aroma regulation of *E. sinensis*. The combination of hedonic and just-about-right (JAR) scales is widely applied in the food industry to provide directional information for reformulation [10,11]. However, the application of these methods is still scarce when evaluating the quality of the flavor of *E. sinensis*.

The factors influencing the odor profile of *E. sinensis* (varieties, breeding environment, gender, edible parts) and consumer preference remain unclear. Such studies could effectively optimize the sensory quality of aquatic products. Therefore, this study aimed to investigate volatiles’ acceptability and optimization potential. We identified compounds of *E. sinensis* from different origins and varieties using monolithic material sorptive extraction (MMSE) combined with gas chromatography-mass spectrometry (GC-MS) analysis. We then combined the volatiles with panel sensory analysis to explore the crab matrix’s crucial compounds that may affect consumer perception.

## 2. Materials and Methods

### 2.1. Materials

Three groups of *E. sinensis* were used: SS (Shanghai, China, Shanghai Baodao Aquaculture Professional Cooperative; varieties: Sea & River 21), TS (Taizhou, China, Taizhou Jiangyuan Animal Husbandry Co., Ltd.; varieties: Sea & River 21), and TC (Taizhou, China, Taizhou Jiangyuan Animal Husbandry Co., Ltd.; varieties: Changjiang 2). TS and TC are two different varieties that were purchased from the same supplier, with the same feeding patterns and rearing conditions. The culture temperature of the three groups was 20.71 ± 1.22 °C. Trash fish and corn were mainly used as feeding in the early stage of culture. The difference was in the fattening stage, which involved snails in Shanghai and pumpkins in Taizhou. A total of 360 *E. sinensis* were collected for the experiment, and their characteristics are listed in Table 1.

The samples were collected in November 2021 and transported to Shanghai Ocean University within 24 h at 4 ℃. The edible parts (crab meat, hepatopancreas, and gonads) were separated to avoid interaction and placed in 0.45 L stew pots and numbered. Room temperature water (4 L)—approximately 20 °C—was placed in a steamer with the pot and steamed at 2000 W for 25 min (including heating the water to 100°C for approximately 10 min).

### 2.2. Experimental Process

#### 2.2.1. Participants

Fifteen panelists (11 females and 4 males, mean age = 23 years) were selected from Shanghai Ocean University according to the ISO standard (ISO, 2007) to assess the intensity of *E. sinensis* odor attributes. First, the panelists discussed the generation of descriptive terms and identified eight odor descriptors (grassy, fatty, metallic, meaty, toasted, ammonia-like, earthy, and fishy). Subsequently, the sensory panel identified the use of a 7-point scale to assess the intensity of each attribute (1 = very weak and 7 = very strong). The definitions of each sensory descriptor are listed in Table S1.

A total of 140 individuals participated in consumer sensory tests. However, for different reasons (such as seafood allergies and a stronger preference for *E. sinensis*), 100 valid results were obtained. The participants were recruited through social media, mainly from Shanghai Ocean University, of which 53/47% were females/males aged between 19–36 years. They were from different regions of China. The participants were given a brief introduction on the use of JAR scales.

Ethical approval for this research was provided by Shanghai Ocean University (approval number: SHOU-DW-2021-085). All participants signed a consent form before participation.

#### 2.2.2. Sensory Evaluation

All experiments were performed under artificial daylight illumination conditions in the professional sensory laboratory of the College of Food of Shanghai Ocean University with a controlled temperature of 22–24 °C. Each sample (3 g)—stored at 20 °C—was placed in a transparent cup with a lid and coded using three random digits. The order of presentation was based on Williams’s Latin square design. All of the participants had a balanced order of attributes. At the beginning of the evaluation, the participants were briefed about the sensory procedures and descriptors. Next, the participants opened the lid and assessed the aroma by agitating the air above the cup to enter the nasal cavity as far as possible. Only one evaluation was allowed in order to avoid fatigue. Sniffing the back of the hand was used as a cleanser to reduce cross-linking effects between samples. The sensory panel completed the attribute intensity assessment, and the participants were required to complete the overall hedonic score and JAR assessment. At the end of the experiment, each participant received a small gift as a reward. The questionnaire was electronic, and the data were collected and exported to the phone background through a questionnaire network (https://www.wenjuan.com, accessed on 3 November 2021). The details of the sensory evaluation were developed according to previous literature (Table 2).

#### 2.2.3. MMSE Combined with GC-MS Analysis

For sample preparation, an aliquot (10 g) of the edible parts was placed into a 40-mL brown headspace vial with a cap (CNW, Shanghai, China), and 5 μL of trimethyl pyridine (purity > 98.0%) was added at a concentration of 10^-7^ g/mL as the internal standard. Next, three Mono Trap TM RCC18 rods were placed at fixed positions in the top space of the vials, equilibrated under continuous stirring for 40 min at 60 °C, and measured using GC-MS.

Furthermore, the sorbent material was removed, loaded into a tube, and transferred to the thermal desorption unit for desorption. Next, the volatiles were condensed through a cold injection system, vaporized at high temperatures, and channeled into the GC for separation. A 7890 A GC system was equipped with a DB-5 MS column (60 m × 0.25 mm ID 1 μm; Agilent Technologies, Basel, Switzerland). The initial temperature was 40 °C (2 min), ramped to 60 °C (2 min), followed by a gradient of 5 °C/min, and finally ramped up to 250 °C (2 min) at the same rate. The injection mode was splitless with a flow rate of 1 mL/min and a vapor chamber temperature of 240 °C. For qualitative assessment, the mass spectra of volatiles were compared with those in the National Institute of Standards and Technology (NIST 2017) and Wiley 9 libraries. A standard mixture of n-alkanes (C_5_–C_30_) was injected into the GC as a sample using the same condition, and retention indices (RI) were calculated.

### 2.3. Statistical Analysis

Each experiment was repeated three times, and the results were shown as mean ± standard deviation (SD). A one-way ANOVA was used for the analysis of significant differences using SPSS v. 24.0 (*p* < 0.05 indicates statistical significance). The drivers of consumer preferences were explored using a stepwise regression analysis. The data showed a non-normal distribution and involved four factors (varieties, origin, gender, and edible parts). The variables of the different factors were explained based on a generalized linear model (GLM). This model uses overall consumer preference as the dependent variable, varieties and origin as independent variables (fixed factors), and edible parts and gender as covariates (main effects and interactions with fixed factors). Additionally, GLM uses “Gamma” as the family function and “Log” as the model link function. 

Mean drops and penalty analyses were performed using the XLSTAT (Addinsoft, New York, NY, USA) statistical software version 2016. Correlations between sensory and volatile compounds were analyzed using the R software version 4.0.3 (RStudio, Inc., Boston, MA, USA).

## 3. Results and Discussion

### 3.1. Intensity of Sensory Attributes

The sensory panel performed quantitative descriptive analyses of all samples to investigate the sensory characteristics of *E. sinensis* (Table 3). Overall, the odor intensity of SS *E. sinensis* was higher than that of Jiangsu, particularly for female crabs. In contrast, the sensory profiles of TS and TC were similar. Among the eight attributes assessed by the participants, fatty, meaty, and earthy odors were significantly different (*p < 0.05*) in female crabs, and males with grassy, metallic, and toasted odors differed from females. Regarding hepatopancreas, the intensity of fatty odor was highest in female crabs in the SS group (5.92), and was significantly higher than that in male and female crabs of the TS and TC groups. Furthermore, the hepatopancreas of males in the TS group was slightly lower at 5.67 and similar to that in the SS and TC groups. Female and male crabs in the SS group had a significantly higher intensity of meaty odor than those in the TS group (6.13 and 6.53, respectively). All attributes of male crab meat were generally higher than those of female meat. In contrast, the intensity of each attribute was lower in the gonads of males than those of females.

Protein and lipid contents in the gonads of female crabs were 1.78 and 18.79 times higher than those of males, especially C16:1 n7 (10.62%) and C18:1 n9 (19.87%) in females, and 3.07% and 14.56% in males, respectively [15]. This may be the underlying reason for the difference in odor profiles produced by crabs of different genders. Most ketones in volatiles are generated by lipid degradation and the Strecker reaction of amino acids [16]. The 2-heptenal (green) and 1-penten-3-one (pungent) in the gonads of male crabs, and 2-butanone (ethereal) and 4-methyl phenol (phenolic) in female crabs may be responsible for this difference [7]. Proteins are predominant in meat, and the fat content is low. Therefore, consumers in these three areas did not differ in their assessment of odor intensity. The odor intensity of male crab gonads was generally low (Table 3) due to a lipid content of approximately 1% wet weight [17]. 

### 3.2. Important Odorants Related to Sensory Attributes

**Figure 1:**
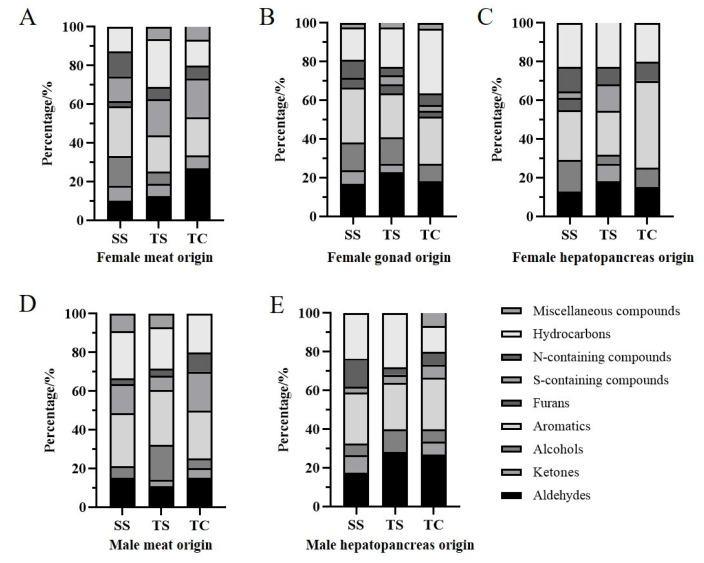
Categories of volatile compounds identified in edible parts of *E. sinensis* from regions. (**A**), (**B**), and (**C**) representing female meats, gonads, and hepatopancreas, respectively. (**D**) and (**E**) represent male meats and hepatopancreas, respectively.

Regarding molecule investigation, the volatiles was identified by MMSE-GC-MS in steamed *E. sinensis*. Specific information on the retention index, odor type, odor description, boiling point, and threshold is provided in Table S2. The 96 volatile compounds are shown in Figure 1.

For crab meat, females in the SS group had 39 compounds with more aromatic compounds (10) than those in the female TS and TC groups (Figure 1A). The number of volatile compounds in SS and TS was broadly similar, except for that in TC; however, SS males contained more aldehydes (5) and sulfur-containing compounds (5) (Figure 1D). Nonanal imparts meaty, fatty, and herbal flavors to crab meat [8]. The compounds such as 3-ethyl-thiophene, 2-propyl-thiophene, 2-butyl-thiophene, and 2, 3-dimethyl-thiophene were found in meat [18]. The 3-methyl-butanal content in female crab meat was significantly higher in the TS group than that in the other groups (376.08 μg/kg in SS group). Furthermore, 3-methyl-butanal and 2-methyl-butanal were identified in female crab meat, with a total concentration 5–6 times higher than that in males (Table S3). 3-Methyl-butanal is a crucial component of crab flavor identified by gas chromatography with olfaction (GC-O) and has been found to have a grassy, fatty, and vegetable-like aroma, originating from lipid degradation, Maillard reaction, and amino acid degradation [19,20]. For female crab meat, benzaldehyde was highest in the TS group at 548.59 μg/kg. It contributes to the sweet odor, as evidenced by olfactory analysis, after being found in seawater crabs [21]. Lastly, 2-acetylthiazole is a heterocyclic flavor compound with a nutty, cereal-like flavor that emits a popcorn-like aroma with a low threshold (10 μg/kg). Female crab meat contained more 2-acetythiazole than males in the TS (510.31 μg/kg) and TC groups (272.05 μg/kg). In contrast, male meat contained more alkane. The content in males was significantly lower than that in females, consistent with a previous study [22].

The odor profile of the gonads of female crabs was unique compared with that of males (Figure 1B). The highest content of 1-octen-3-ol (170.77 μg/kg) was found in the gonads of the SS group; it often imparts a mushroom and fishy aroma. Furthermore, it is derived from the oxidative decomposition of linoleic acid and is present in the hepatopancreas [23]. Additionally, nitrogen/sulfur-containing compounds contribute to the flavor of *E. sinensis*. Trimethylamine is the only nitrogenous compound with an odor activity value (OAV) > 10 and is produced by trimethylamine oxides through microbial metabolism [24]. Trimethylamine was detected in the edible parts from all three areas, and its concentrations were generally higher in females than in males, especially in the gonads of SS. High concentrations of trimethylamine are undesirable in seafood; however, it may impart a pleasant crustacean odor at low concentrations [25].

The hepatopancreas is the main organ for lipid metabolism in *E. sinensis* and has a high crude fat content [26]. Color and flavor are two important sensory attributes for adult crabs with a high hepatopancreas index. For females, the SS group had the highest number of volatile compounds (31) in the hepatopancreas, with a rich variety. Benzaldehyde—1422.98 μg/kg in the SS group—is considered an odor-active compound in the hepatopancreas and gonads, presenting a bitter almond odor [8]. The SS female group had the highest content of 2-acetylthiazole at 457.80 μg/kg, imparting a unique and pleasant odor profile [22]. The C-4 and C-5 atoms of 2-acetylthiazole are derived from amino acids in the Maillard reaction involving sugar and cysteine/cysteamine [27]. Compared with females, the types and composition of volatiles in males were similar; however, the content of each compound in males was generally lower than in females.

**Figure 2:**
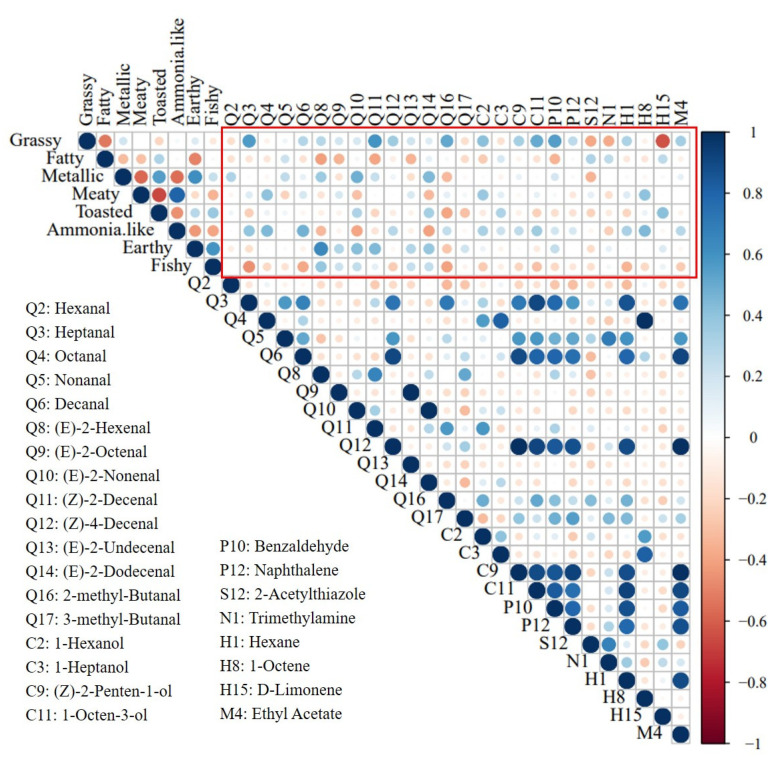
Correlation analysis of volatile compounds and sensory attributes in *E. sinensis*. The data in the figure are active odor compounds identified as OAV > 10 from the three origins. Positive correlations are marked in blue, negative in red, and the correlations enhance with the deepening of the color.

The relationship between volatile compounds and sensory attributes in *E. sinensis* was assessed using a correlation analysis (Figure 2). We observed positive correlation between 2-acetylthiazole and fatty odor, whereas the opposite was observed for (E)-2-hexenal and (E)-2-octenal. For earthy and fishy odors, (E)-2-hexenal, (E)-2-nonenal, and (Z)-2-decenal showed strong positive correlations. Furthermore, heptanal, (Z)-2-decenal, 2-methyl-butanal, 1-octen-2-ol, and benzaldehyde were positively correlated with grassy odors. However, the contribution of volatiles to the odor profile of *E. sinensis* was limited; and it was based solely on concentration and threshold in the air. The different nutrient contents in foods (fats, proteins, and carbohydrates) can significantly affect the release of volatiles [28]. Several flavor improvement and sensory acceptance studies have shown that appropriate concentrations of aromatic compounds may result in desirable flavors, whereas an excess can reduce acceptance [29]. Notably, interactions (for example, synergistic effects) may occur between different odorants, altering their characteristics. Therefore, whether the above sensory intensity and volatile compounds affect consumer preferences needs to be further explored.

### 3.3. Overall Consumer Preferences in E. sinensis

**Figure 3:**
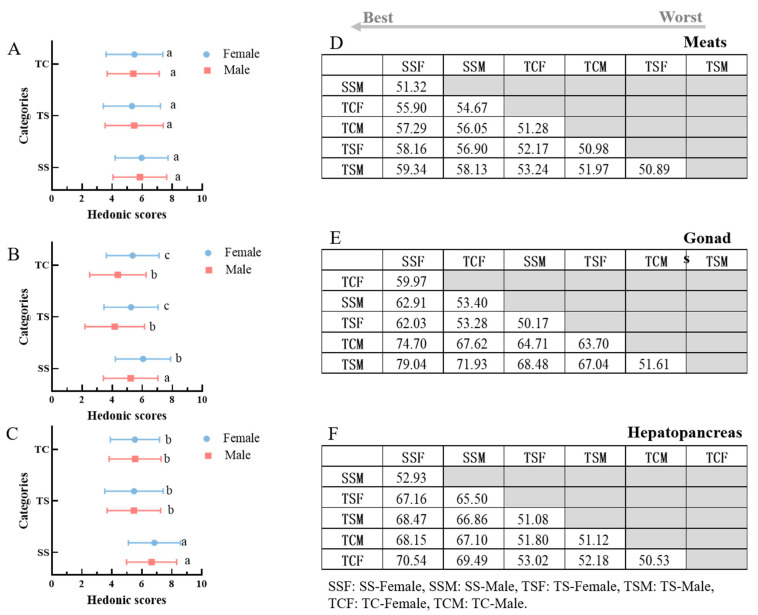
Overall consumer preference assessment of the odor profile for *E. sinensis* from three regions (N = 100). (**A,B,** and **C**) represent the mean and significance calculated using traditional ANOVA/LSD statistical analysis. (**D,E,** and **F**) are R-index values (R_MAT_) calculated from the ranking data derived from the nine-point hedonic scale responses. (**A** and **D)** represent data from crab meat, (**B** and **E**) represent data from gonads, and (**C** and **F**) represent data from the hepatopancreas. The R_MAT_ index indicates a preference for the samples in the first row over the left column. For example, 51.32% of consumers in Figure 1D prefer I to II. A higher R_MAT_ value indicated a more significant difference between the two samples. The first row of samples, arranged from left to right, indicates the preference from best to worst. Significant differences are indicated by lowercase letters (a, b and c).

Consumers’ overall hedonic scores for the odor profiles of edible parts in *E. sinensis* from three regions was analyzed using traditional significance analysis (Figure 3A–C). To further analyze the strength of the overall preference among the samples, the R-index (R_MAT_) was introduced to compute the preference effect size (Figure 3D–F). The variables were similar and conversely different when R_MAT_ values were approximately 50%. The effects of four factors (variety, breeding origin, gender, and edible parts) on consumer preferences are discussed separately. The results of the R_MAT_ values were consistent with the traditional statistical analysis method for significant differences, and we quantified the degree of difference between consumer preferences.

Regarding the varieties factor, the SS group was the most popular among consumers. However, there was no significant difference between TS and TC, located in Jiangsu, with R_MAT_ values of approximately 50%. The hepatopancreas and gonads from Shanghai were significantly higher than those from Jiangsu by approximately 1.03–1.25 times. For example, SS-Female (SSF) was preferred to TS-Female (TSF) by 67.16% of the consumers. Regarding the gender factor, the R_MAT_ indices of meat were below 60%, with little difference between samples (Figure 3D). The gonads were significantly higher in females than those in males by approximately 1.15–1.25 times. Furthermore, 62.91% of consumers preferred SSF to SSM (Figure 3E). During handling and storage, the higher free amino acids (FAAs) content in the hepatopancreas of females may oxidize more readily compared with that of males to produce volatile compounds [30]. However, an ANOVA/LSD analysis showed no significant difference between SSF and SSM in the hepatopancreas, with an R_MAT_ value of 52.93% (Figure 3). Regarding the edible part factor, the hepatopancreas was most preferred by consumers and was the dominant source of crab flavor, followed by gonads. The mean value for female gonads was 5.25–6.05, significantly higher than that for males. 

### 3.4. Drivers of Consumer Preferences

The taste of the crab, especially the umami and sweetness, is the crucial factor driving consumption. It is worth mentioning that the attractive color and intense characteristic volatiles formed the first impression of a cooked crab. Besides appearance, the aroma creates emotional responses and significant food memories that influence consumer decisions [31]. External (rearing origin) and internal factors (varieties, gender, and edible parts) can change the nutrient composition of *E. sinensis*, resulting in different flavors. Therefore, a GLM was performed using stepwise multiple regression analysis to investigate the contribution of the four factors (varieties, origin, gender, and edible parts) to consumers’ overall aroma preference for *E. sinensis* (Table 4).

According to the stepwise regression model of overall consumer preference in the literature, the explanatory rate of the following model is valid [32]. Stepwise regression methods, including backward and forward-backward regression, were significantly different (*p < 0.05*), and the goodness of fit in backward regression was superior to that in forward-backward regression. The stepwise regression GLM is provided in Table 4, where the origin, gender, and edible parts variables were significant (*p < 0.05*). No significant correlation was observed between varieties and consumer preference. The variables in the backward regression explained 79.2% of the variance in the total score (R^2^ = 0.792), whereas the regression results of the forward-backward method explained 79.7% of the variance (R^2^ = 0.797). Specifically, the variance in females (gender: male, coefficient = –0.068, Std. Error = 0.029, *p = 0.035*), Shanghai origin (origin: Jiangsu, coefficient = –0.151, Std. Error = 0.03, *p < 0.001*), and hepatopancreas (parts: meat, coefficient = –0.055, Std. Error = 0.035, *p = 0.144*; parts: gonad, coefficient = –0.156, Std. Error = –0.035, *p < 0.001*) were aligned with significantly higher consumer hedonic scores.

Variety was not a significant factor influencing consumer preference for the odor profile of *E. sinensis* (*p > 0.05*); therefore, we excluded it from the forward-backward regression model discussion. However, differences between origins significantly affected consumers’ preferences. The factor "origin" probably relates to organisms in the environment [33]. Low biodiversity of microalgae and flourishing macrophytes could directly or indirectly affect the quality of *E. sinensis* flavor [34]. In addition, *E. sinensis* of multiple origins have different feeding and management patterns. However, the patterns of the crab’s daily feeding are crucial for its quality. Feeding plant- or animal-derived feeds at the same growth stage could cause differences in intestinal flora to affect nutrient absorption and metabolism [35]. The accumulation of nutrient substances (such as lipids) in the crab is also the main reason for the unique odor characteristics.

The relationship between inherent attribute variables (gender and edible parts) and consumer preference for *E. sinensis* may be explained by differences in physicochemical composition. Given the high concentration of lipids in the gonads and hepatopancreas, steamed *E. sinensis* releases aromas that are preferable among consumers. The total lipid content of the hepatopancreas of female crabs was approximately 47.11% wet weight and that in males was 41.22% wet weight [36]. Monounsaturated fatty acids, especially oleic acid, are associated with the aroma produced by the thermal oxidation of lipids. Additionally, the male crab gonads contained almost no lipids (only 0.67%), whereas females contained approximately 20 times the total lipids of males, which may be the primary reason for the difference in consumer preference for the odor profile between male and female crabs. The fat content was only 1.04% wet weight, with little difference between male and female crabs [37]. Therefore, the parts of meat did not contribute significantly to consumer preference in the GLM stepwise regression model.

In summary, the varieties of *E. sinensis* was not the primary factor, and origin, gender, and edible parts significantly affected consumer preferences. 

### 3.5. Acceptance of Sensory Attributes Relative to Consumers’ Ideal Level

**Figure 4:**
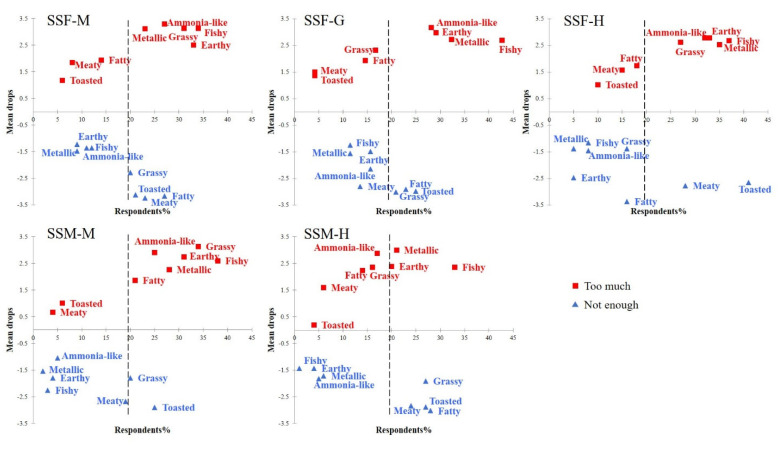
Just-about-right (JAR) percentages of consumer response for sensory attributes in three edible parts of *E. sinensis* (female/male) from Shanghai (n = 96). The mean drops indicate that the extent of the overall consumer preference decreases when the attribute’s intensity deviates from the ideal. M: crab meat, G: gonads, H: hepatopancreas.

A commodity is futile if not consumed or liked by consumers, even if it has outstanding nutritional and physiological properties. Therefore, determining the flavor attributes that affect consumer preferences is essential in food development and optimization. Taking the SS group as an example, the acceptability of each attribute intensity of *E. sinensis* to consumers was assessed using a 5-point scale (Figure 4). All the attributes discussed are listed in Table 4. The mean drop represents a declining score in consumer preference due to this effect. Penalty analysis can verify which sensory attributes affect the overall preference. For the edible parts (meat, gonads, and hepatopancreas), the responses of consumers who considered the intensity of sensory attributes to be just- about- right was 46–82% for females and 46–78% for males in the SS group (Figure S1). According to the thresholds described in the reported studies, a significant effect was considered when the remaining respondents were >20%, and the mean drop was > or = 1 [38].

Consistent with the overall preference results, female and male crabs exhibited similar results, except for the gonads. Ammonia-like (mean drop = 3.30/2.90), grassy (mean drop = 3.13/3.13), metallic (mean drop = 3.11/2.26), earthy (mean drop = 2.50/2.74), and fishy odor (mean drop = 3.13/2.59) were the main factors that decreased preference for crab meat, as shown in Figure 4A,D. Adding toasted flavor (mean drop = –3.11/–2.91) could significantly enhance consumer enjoyment. Since the gonads of females have an abundant odor profile, they are more likely to evoke positive emotions than males. Ammonia-like, earthy, metallic, and fishy odor in the gonads resulted in lower preferences, and >25% of consumers held this view (Figure 4B). Notably, the consumers preferred gonads with higher fatty (mean drop = –2.91) and baked flavors (mean drop = –2.98) (Figure S1). As with the gonads, the increasing fatty, meaty, and toasted flavor in the hepatopancreas may improve preference (Figure 4C,E). Interestingly, high concentrations of these attributes had little effect on preference. It is speculated that as the intensity of some sensory characteristics increases, favorability improves rapidly at first and then levels off or declines.

Regarding the *E. sinensis* odor profile, the JAR results highlight sensory attributes that interest most consumers. The most important sources of crab flavor in *E. sinensis*, especially in males, are the hepatopancreas and gonads. The hepatopancreas is rich in n-3 unsaturated fatty acids, essential fatty acids, essential amino acids, and vitamins; hence, it is a highly nutritious aquatic food [15]. Polyunsaturated fatty acids (PUFA) contribute significantly to crabs’ characteristic flavor by producing aldehydes, alcohols, ketones, or other compounds through oxidation [39]. Further findings indicate that the ’Fatty’ in the hepatopancreas may be the main reason for the differences in odor profiles of crabs from different breeding regions and would significantly affect overall consumer preference. The appropriate application of meaty and roasted flavors in the hepatopancreas may improve consumer preferences. A possible reason is that the typical meat flavors, nonanal (fatty, citrus) and decanal (citrus, floral), often evoke positive emotions in consumers [40]. The nutritional composition of male and female crab meat is similar; however, PUFA, especially n-3 PUFA, is higher in males than in females [41]. The thermal oxidative decomposition of linolenic acid, EPA, and DHA produces volatile compounds, such as propanal (earthy, nutty), 2-hexenal (fatty, cheesy), 2, 4-heptadienal (pungent, fruity), and 2, 6-nonadienal (fatty, cucumber) [42]. The additional odor characteristics imparted by these compounds may be the reason for the preference for male crab meat compared to female crab meat. Therefore, the complete crab flavor profile in consumers’ memories may be a combination of the hepatopancreas and the meat aroma. The appropriate meat odor could optimize the aroma of crab derivatives.

## 4. Conclusions

For all samples, the odor of the hepatopancreas was the most preferred among consumers, especially that of female crabs. Furthermore, >52.93% of consumers preferred the hepatopancreas odor profile of female crabs in Chongming, with an overall preference of 6.84 and a fatty intensity of 5.42. The primary reason for the different odor profiles of the crabs was their origin rather than the varieties. The appropriate meaty odor and toasted odor in the hepatopancreas would increase the overall preference by approximately 14.65–20.60%. Additionally, not all compounds with fatty odors contributed significantly to the odor profile of *E. sinensis*. Furthermore, 2-acetylthiazole showed a significant positive correlation with fatty odor, whereas the opposite was observed for (E)-2-hexenal and (E)-2-octenal. These findings have potential implications for understanding consumer preferences, affecting the consumption behavior of *E. sinensis*. Moreover, identifying the odorous compounds associated with preference can improve targeted overall flavor in the future and provide theoretical guidance for the aquaculture industry.

## Figures and Tables

**Table 1:** The characteristics of *E. sinensis*.

Code	Samples	Weight/kg	Width/mm	Length/mm	Gonad index(**♀**)/%	Gonad index(**♂**)/%
SS *	60 females, 60 males	136.10 ± 10.57 ^a^	69.08 ± 2.11 ^a^	64.00 ± 1.86 ^a^	9.82 ± 0.36 ^a^	2.54 ± 0.35 ^a^
TS *	60 females, 60 males	137.80 ± 13.81 ^a^	68.27 ± 2.55 ^a^	63.64 ± 1.32 ^a^	8.67 ± 1.44 ^ab^	1.89 ± 0.43 ^b^
TC *	60 females, 60 males	128.93 ± 13.10 ^a^	65.98 ± 2.64 ^a^	61.01 ± 3.20 ^a^	7.72 ± 1.27 ^b^	1.63 ± 0.33 ^b^

* Sample name interpretation. SS = Shanghai Baodao Aquaculture Professional Cooperative, Sea & River 21; TS = Taizhou Jiangyuan Animal Husbandry Co., Ltd., Sea & River 21; TC = Taizhou Jiangyuan Animal Husbandry Co., Ltd., Changjiang 2. ^a,b^ Significant differences are indicated by superscripted lowercase letters.

**Table 2:** Sensory evaluation process and description of *E. sinensis*.

Methods	Description	References
Hedonic Rating	9-point: 1 = “dislike extremely”, 9 = “like extremely” R_MAT_ value measured the strength of preference for overall hedonic scores, providing the percentage of consumers who preferred one sample over another.	[12]
Intensity Evaluation	7-point: 1 = “extremely weak”, 7 = “extremely strong”. Sensory descriptors, including grassy, fatty, metallic, meaty, toasted, ammonia-like, earthy, and fishy.	[13]
Just-About-Right Scale	5-point: 1 = “not enough”, 3 = “just about right”, 5 = “too much”.	[14]

**Table 3:** Comparison of attributes used to describe three edible parts (meat, gonads, and hepatopancreas) of *E. sinensis*.

Attributes	SS			TS			TC		
	Meat	Gonad	Hepatopancreas	Meat	Gonad	Hepatopancreas	Meat	Gonad	Hepatopancreas
**Female**									
Fatty *	4.67 ± 0.72 ^a^	5.73 ± 1.67 ^bdA^	5.93 ± 0.70 ^b^	3.67 ± 0.82 ^cB^	4.80 ± 1.70 ^a^	4.87 ± 0.64 ^adB^	4.80 ± 1.93 ^a^	4.80 ± 1.47 ^aA^	5.40 ± 0.91 ^ab^
Meaty *	6.13 ± 0.64 ^a^	4.47 ± 1.60 ^bA^	4.53 ± 1.60 ^bA^	4.80 ± 0.68 ^bcB^	4.53 ± 1.88 ^b^	4.00 ± 1.93 ^b^	5.80 ± 0.56 ^ac^	4.33 ± 1.91 ^bA^	3.60 ± 1.84 ^bB^
Earthy *	2.20 ± 1.08 ^b^	3.67 ± 0.72 ^ac^	2.87 ± 1.88 ^abB^	3.00 ± 1.69 ^ab^	4.07 ± 1.62 ^a^	3.27 ± 1.62 ^ab^	2.40 ± 1.30 ^bcB^	3.33 ± 1.11 ^ab^	3.00 ± 1.89 ^abA^
Grassy	4.07 ± 1.39 ^A^	3.60 ± 0.74	4.13 ± 1.46	4.53 ± 0.74	3.73 ± 1.22 ^B^	3.87 ± 1.73 ^B^	4.47 ± 1.92	4.80 ± 1.90 ^A^	4.07 ± 1.58
Metallic	2.33 ± 1.54	3.13 ± 1.96	2.80 ± 1.26	3.13 ± 2.03 ^A^	3.47 ± 0.92	3.00 ± 1.73	3.00 ± 1.46 ^A^	3.53 ± 1.36	3.20 ± 1.21 ^A^
Toasted	3.73 ± 1.16	3.87 ± 1.77 ^A^	3.67 ± 1.59 ^A^	3.27 ± 1.67 ^B^	3.67 ± 1.84	3.47 ± 1.36	4.07 ± 1.62	4.20 ± 2.14 ^A^	3.13 ± 1.60
Ammonia-like	2.27 ± 0.80	2.87 ± 0.83 ^B^	2.67 ± 1.88	3.13 ± 1.92	2.73 ± 1.10	2.87 ± 1.55	2.27 ± 1.79	2.93 ± 0.96	3.47 ± 1.46 ^A^
Fishy	3.47 ± 0.74	3.60 ± 0.83	3.60 ± 1.45 ^B^	3.80 ± 0.86 ^B^	3.47 ± 1.73	4.40 ± 2.23	4.20 ± 2.18	3.67 ± 1.45 ^B^	4.33 ± 1.40 ^A^
**Male**									
Fatty *	4.60 ± 1.80 ^ab^	3.33 ± 0.90 ^bB^	5.27 ± 1.58 ^a^	4.53 ± 1.77 ^abA^	4.13 ± 1.68 ^ab^	5.67 ± 1.59 ^aA^	4.40 ± 2.10 ^ab^	3.33 ± 0.72 ^bB^	5.33 ± 1.59 ^a^
Meaty *	6.53 ± 1.41 ^a^	2.93 ± 0.96 ^cB^	3.73 ± 0.88 ^bcB^	6.20 ± 2.14 ^aA^	4.07 ± 2.15 ^bc^	3.80 ± 1.74 ^bc^	5.40 ± 1.84 ^ab^	3.47 ± 1.36 ^cB^	4.20 ± 1.42 ^bcA^
Grassy *	3.33 ± 0.82 ^bB^	3.67 ± 0.98 ^ab^	3.67 ± 1.11 ^ab^	4.00 ± 1.31 ^ab^	4.20 ± 0.94 ^abA^	5.07 ± 0.80 ^aA^	4.20 ± 2.04 ^ab^	3.60 ± 1.68 ^abB^	3.80 ± 1.32 ^ab^
Metallic *	2.40 ± 0.63 ^b^	3.47 ± 0.74 ^ab^	3.20 ± 1.15 ^ab^	2.27 ± 1.16 ^bB^	2.93 ± 1.22 ^ab^	3.33 ± 2.06 ^ab^	2.40 ± 1.72 ^bB^	4.00 ± 1.36 ^a^	2.60 ± 0.91 ^abB^
Toasted *	4.20 ± 1.78 ^ac^	2.73 ± 1.28 ^bB^	3.07 ± 2.19 ^abB^	4.80 ± 1.26 ^cA^	3.13 ± 1.55 ^ab^	3.53 ± 1.55 ^ab^	3.53 ± 1.88 ^ab^	2.80 ± 1.52 ^bB^	3.60 ± 1.12 ^abc^
Ammonia-like	2.73 ± 0.88	3.40 ± 0.99 ^A^	2.87 ± 1.88	3.00 ± 1.51	2.53 ± 1.51	2.73 ± 1.03	2.53 ± 1.30	2.93 ± 0.59	2.47 ± 1.81 ^B^
Earthy	2.80 ± 1.21	3.40 ± 1.40	3.67 ± 1.63 ^A^	3.20 ± 1.90	3.60 ± 0.83	3.93 ± 2.28	3.07 ± 1.91 ^A^	3.73 ± 0.88	2.40 ± 1.55 ^B^
Fishy	4.20 ± 1.47	4.07 ± 1.44	4.47 ± 1.77 ^A^	4.53 ± 1.92 ^A^	3.80 ± 1.90	4.27 ± 1.83	4.33 ± 0.98	4.40 ± 0.83 ^A^	3.40 ± 0.83 ^B^

Significant sensory attributes are indicated by asterisks. Significant differences between edible parts and origins are indicated by superscripted lowercase letters. Differences between genders are indicated by capital letters.

**Table 4:** Stepwise multivariable regression analysis for the overall consumer preference for *E. sinensis* from different origins.

Items	Backward Regression				Forward-Backward Regression			
	Coefficient	Std. Error	*p*-Value	R^2^	Coefficient	Std. Error	*p*-Value	R^2^
Varieties Changjiang 2	0.019	0.036	0.617	0.792	-	-	-	0.797
Origin Jiangsu	–0.160	0.036	< 0.001 ***		–0.151	0.030	<0.001 ***	
Gender Male	–0.068	0.030	0.041 *		–0.068	0.029	0.035 *	
Edible Parts Meat	–0.055	0.036	0.158		–0.055	0.035	0.144	
Gonad	–0.156	0.036	0.001 **		–0.156	0.035	<0.001 ***	

Std. Error means Standard Error. Only one or two variables for each factor are listed. The remaining variables were used as the criteria. Variables not listed in the table as reference variables include Sea & River 21 (varieties), Shanghai (origin), female (gender), and hepatopancreas (parts). * indicates significant differences between variables (*p* < 0.05), ** indicates significant differences between variables (*p* < 0.01), *** indicates significant differences between variables (*p* < 0.001)

## Data Availability

The data presented in this study are available in the article.

## Supplementary Materials

The following supporting information can be downloaded at https://www.mdpi.com/article/10.3390/ani13030541/s1, Figure S1: Percentage of Just-About-Right (JAR) consumers’ responses to the overall preference for *E. sinensis* in Shanghai (n = 100); A, B, and C represent the meat, gonads, and hepatopancreas for females, respectively. C and D represent the meat and hepatopancreas of males, respectively; Table S1: Sensory descriptors and definitions for each descriptor; Table S2: Retention index, boiling points, odor types, odor descriptions, and thresholds of volatile compounds identified in the meat, gonads, and hepatopancreas of *E. sinensis* from three areas; Table S3: The concentrations of volatile compounds were identified in the edible parts of *E. sinensis* from three areas (μg/kg, n = 3); Table S4: Active aromatic compounds (OAV > 10) in the edible parts of *E. sinensis* from three areas.
