# Supplementary material for: Drivers of Consumer Preference Derived from Active Volatiles for Cooked Eriocheir sinensis
Source: Animals (Basel). 2023 Feb 3;13(3):541. doi: 10.3390/ani13030541 (PMC9913383; doi:10.3390/ani13030541)
Supplement: Supplementary file 1 [file animals-13-00541-s001.zip › animals-2108647-supplementary.pdf]

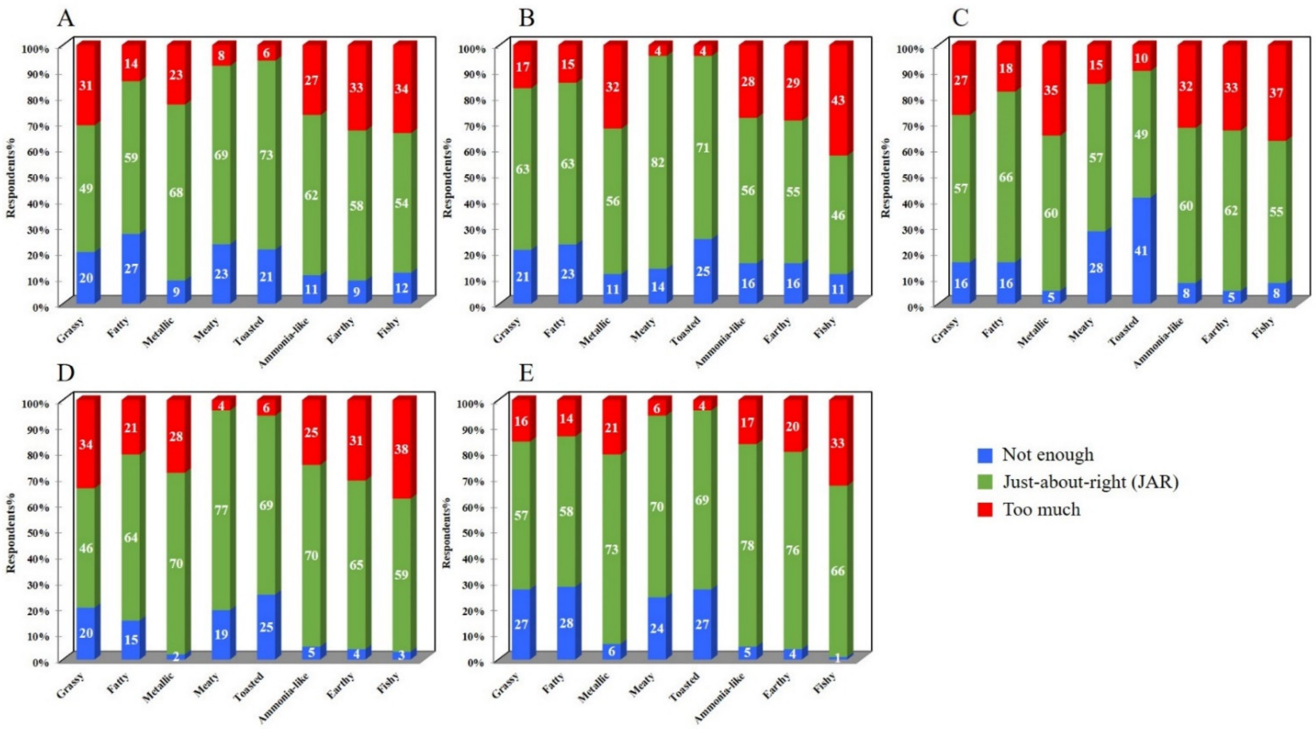

Figure S1. Percentage of Just-About-Right (JAR) consumers' responses on overall preference for *E. sinensis* in Shanghai (n=100) A, B, and C represent the meat, gonads, and hepatopancreas for females, respectively. D and E represent the meat and hepatopancreas for males, respectively.

Table S1. Sensory descriptors and definitions for each descriptor.

No.	Descriptors	Definitions
1	Grassy	Aromatics associated with mowed lawn
2	Fatty	The aromatics resulting from the natural deterioration of a food rendered fat.
3	Metallic	(1) The aromatic associated with metals, tinny or iron;<br>(2) A flat feeling factor stimulated on the tongue by metal.
4	Meaty	Aromatics associated with cooked meat
5	Toasted	Aromatics associated with baked goods, combined with caramel, sweet and milky
6	Ammonia-like	Aromatics associated with ammonia, slightly irritant
7	Earthy	Flavor associated with soil
8	Fishy	Flavor associated with cooked white fish

Table S2. Retention index, boiling points, odor types, odor descriptions, and thresholds of volatile compounds identified in meat, gonads, and hepatopancreas of *E. sinensis* from three areas.

Code	RI [a]	Compounds	Boiling point（℃）	Odor Type [b]	Odor Description [c]	Threshold [c] (μg/kg)
		**Aldehydes (21)**				
Q1	699	Pentanal	103	Fermented	bready, fruity, nutty	9
Q2	800	Hexanal	129.6	Green	green, grassy, fatty	5
Q3	901	Heptanal	153	Green	fresh, aldehydic, fatty	2.8
Q4	1004	Octanal	163.4	Aldehydic	Fatty, pungent, waxy, citrus, orange, herbal, fresh	0.587
Q5	1104	Nonanal	191	Aldehydic	waxy, fatty, orange	1.1
Q6	1206	Decanal	207-209	Aldehydic	sweet, waxy, citrus	0.1
Q7	754	(E)-2-Pentenal	126.8	Green	pungent, green, fruity	199.69
Q8	873	（E)-2-Hexenal	146-149	Green	green, banana, fatty	19.2
Q9	854	(E)-2-Octenal	84-86	Fatty	fatty	3
Q10	1157	（E)-2-Nonenal	189	Fatty	fatty, green, cucumber	0.08
Q11	1263	(Z)-2-Decenal	229	Fatty	waxy, earthy, green	0.3
Q12	1193	(Z)-4-Decenal	200.06	Citrus	orange, aldehydic, watery	0.02
Q13	1365	（E)-2-Undecenal	229	Fruity	fruity, citrus	0.78
Q14	1482	（E)-2-Dodecenal	93	Herbal	citrus, metallic, mandarin	1.4
Q15	1012	(E, E)-2,4-Heptadienal	177.4	Fatty	fatty, green, oily, aldehydic	15.4
Q16	663	2-methyl-Butanal	90-92	Cocoa	musty, cocoa, coffee, nutty	1
Q17	670	3-methyl-Butanal	93.5	Aldehydic	ethereal, chocolate, fatty	1.1
Q18	745	(E)-2-methyl-2-Butenal	115	N.A.	N.A.	458.9
Q19	762	2-ethyl-Butanal	136.5	Green	sweet, green, ethereal	-
Q20	956	2-ethyl-Hexanal	184.6	N.A.	N.A.	125
Q21	1365	2-methyl-Undecanal	171	Aldehydic	fresh, amber, mossy, citrus	-
		**Ketones (8)**				
T1	573	2-Butanone	76.9	Ethereal	fruity, camphoreous	35400.2
T2	990	2-Octanone	173.5	Earthy	earthy, weedy, natural, woody	50.2
T3	1092	2-Nonanone	195.3	Fruity	fresh, sweet, green	38.9
T4	1294	2-Undecanone	228	Fruity	waxy, fruity, creamy, fatty	5.5
T5	1396	2-Dodecanone	247.8	Citrus	fatty	42
T6	1497	2-Tridecanone	263	Waxy	fatty, waxy, dairy, coconut	-

T7	986	6-methyl-5-Hepten-2-one	173.28	Citrus	green, musty, citrus	68
T8	865	5-methyl-2-Hexanone	145	N.A.	N.A.	62
		**Alcohols (14)**				
C1	765	1-Pentanol	136-138	Fermented	fusel, oily, sweet	150.2
C2	874	1-Hexanol	157	Herbal	ethereal, fusel, oily	5.6
C3	970	1-Heptanol	176.45	Green	musty, leafy, violet, herbal	5.4
C4	998	2-Octanol	178	Spicy	fresh, spicy, green, woody	7.8
C5	1102	2-Nonanol	195.5	Waxy	green, creamy, citrus, orange	58
C6	1298	2-Undecanol	228	Waxy	fresh, waxy, cloth	8.6
C7	684	1-Penten-3-ol	114-115	Green	ethereal, green, radish	358.1
C8	725	3-Penten-1-ol	140	N.A.	N.A.	-
C9	767	(Z)-2-Penten-1-ol	141.3	Green	green, phenolic, ethereal	89.2
C10	732	5-Hexen-2-ol	139	N.A.	N.A.	-
C11	980	1-Octen-3-ol	175	Earthy	mushroom, earthy, green	1.5
C12	1257	(E)-2-Decen-1-ol	230.3	Fatty	waxy, ozone, citrus, rose	-
C13	1031	2-ethyl-1-Hexanol	183-186	Citrus	fresh, floral, oily	1500
C14	1028	2-methyl-1-Hexadecanol	318	N.A.	N.A.	-
		**Aromatics (12)**				
P1	654	Benzene	80.1	N.A.	N.A.	3630
P2	763	Toluene	110.6	N.A.	N.A.	1550
P3	855	Ethylbenzene	136.2	N.A.	N.A.	2205.25
P4	865	p-Xylene	138.3	N.A.	N.A.	530
P5	887	o-Xylene	143	N.A.	N.A.	450.23
P6	893	Styrene	145.2	Balsamic	sweet, floral, balsamic	65
P7	953	Propylbenzene	160.51	N.A.	N.A.	177.12
P8	1054	Butylbenzene	183	N.A.	N.A.	100
P9	1157	Pentylbenzene	205	N.A.	N.A.	-
P10	962	Benzaldehyde	179	Fruity	sweet， almond	41.7
P11	990	1,2,4-trimethyl-Benzene	168	N.A.	N.A.	
P12	1182	Naphthalene	217.9	Pungent	pungent, dry, resinous	60
		**Furans (3)**				
F1	614	3-methyl-Furan	65	N.A.	N.A.	-
F2	833	Furfural	161.7	Bready	sweet, woody, almond	9562
F3	859	2-Furanmethanol	171	Bready	musty, sweet, alcoholic	4500.5
		**S-containing compounds (12)**				

S1	616	Propyl mercaptan	67	Alliaceous	cabbage, gassy, sweet, onion	3.1
S2	1233	1-Nonanethiol	219.97	N.A.	N.A.	-
S3	746	Dimethyl disulfide	109	Sulfurous	vegetable, cabbage, onion	12,3
S4	824	Dimethyl Sulfoxide	189	Alliaceous	fatty, oily, cheesy	-
S5	970	Dimethyl trisulfide	41	Alliaceous	meaty, onion, savory	-
S6	775	2-methyl-Thiophene	107.9	Sulfurous	alliaceous, onion, roasted, green	-
S7	869	3-ethyl-Thiophene	138.31	Styrene	styrene	-
S8	961	2-propyl-Thiophene	-	Chemical	chemical	-
S9	1069	2-butyl-Thiophene	181	Fruity	floral, milky, fried, chicken	-
S10	896	2,3-dimethyl-Thiophene	-	N.A.	N.A.	-
S11	735	Thiazole	-	Fishy	fishy, nutty, meaty	38
S12	1022	2-Acetylthiazole	212.5	Popcorn	nutty, popcorn, peanut	10
		**N-containing compounds (7)**				
N1	502	Trimethylamine	2.87	Fishy	fishy, oily, rancid	2.4
N2	755	Pyrrole	130	Nutty	sweet, ethereal	20000
N3	746	Pyridine	115.3	Fishy	sour, fishy, ammoniacal	2000
N4	863	3-methyl-Pyridine	144	Green	earthy, hazelnut, nutty	-
N5	853	4-methyl-Pyrimidine	-	N.A.	N.A.	-
N6	1027	3-propyl-Pyridine	-	Beany	sweet, green, musty, earthy	-
N7	1101	3-butyl-Pyridine	-	N.A.	N.A.	-
		**Hydrocarbons (15)**				
H1	600	Hexane	69	N.A.	N.A.	1500
H2	700	Heptane	98	N.A.	N.A.	50000
H3	800	Octane	125-127	N.A.	N.A.	10000
H4	900	Nonane	151.66	N.A.	alkane, gasoline	10000
H5	1100	Undecane	196.28	N.A.	N.A.	1170
H6	685	1-Heptene	94	N.A.	N.A.	-
H7	691	(Z)-3-Heptene	96	N.A.	N.A.	-
H8	789	1-Octene	121	N.A.	N.A.	0.5

H9	810	2-Octene	125.2	N.A.	N.A.	-
H10	889	1-Nonene	146.9	N.A.	N.A.	-
H11	885	cis-4-Nonene	143	N.A.	N.A.	-
H12	989	1-Decene	169	N.A.	N.A.	-
H13	1097	(E)-2-Undecene	-	N.A.	N.A.	-
H14	523	1,3-Pentadiene	42	N.A.	N.A.	2500
H15	1018	D-Limonene	176	Citrus	orange, fresh, sweet	10
		**Miscellaneous compounds (5)**				
M1	700	Propanoic acid	141.1	Acidic	pungent, acidic, cheesy	2190
M2	1273	Nonanoic acid	255.6	Waxy	waxy, dirty, cheesy, dairy	4600
M3	1475	Undecanoic acid	283.3	Waxy	creamy, cheesy, fatty	10000
M4	612	Ethyl Acetate	77.2	Ethereal	fruity, sweet, weedy	5
M5	1045	2-Heptanol, acetate	-	Brown	fatty, fruity, green	-

N.A. means not available.

a Retention index on DB-5MS column (compared with the RI in the literature).

b Odor types are mainly gathered from the website database. (http://www.thegoodscentscompany.com/index.html)

c Odor descriptions and thresholds are mainly gathered from literature (Gu, Wang, Tao, & Wu, 2013) (Wu, Gu, Tao, Wang, & Ji, 2014) (Wang et al, 2016) (Wu, Wang, Tao, & Ni, 2016) (Zhuang et al, 2016)

Table.S3. The concentrations of volatile compounds were identified in edible parts of *E. sinensis* from three areas (μg/kg, n=3).

Compounds	Female-M			Female-G			Female-H			Male-M			Male-H		
	SS	TS	TC	SS	TS	TC	SS	TS	TC	SS	TS	TC	SS	TS	TC
**Aldehydes (20)**															
Pentanal	-	-	-	-	-	67.90±1.80^{a}	-	-	-	-	-	-	-	63.02±3.07^{a}	-
Hexanal	-	-	17.70±0.02^{c}	-	285.12±24.27^{ab}	96.38±5.09^{abc}	-	295.58±2.01^{a}	-	70.62±5.37^{abc}	-	162.35±2.30^{abc}	-	59.59±2.63^{bc}	58.64±0.18^{bc}
Heptanal	-	-	-	124.00±10.16^{a}	37.16±1.50^{b}	-	101.07±2.84^{a}	-	-	-	-	-	-	-	-
Octanal	-	-	-	-	-	63.15±5.43	-	-	-	-	-	-	-	-	-
Nonanal	96.48±2.97^{abc}	99.39±7.03^{c}	20.09±0.65^{c}	271.95±2.24^{a}	79.00±4.26^{c}	80.08±1.13^{bc}	155.20±7.09^{abc}	19.04±1.35^{c}	51.87±4.34^{c}	115.18±7.76^{abc}	96.56±1.89^{abc}	268.60±3.80^{ab}	-	115.30±3.60^{abc}	20.24±0.36^{c}
Decanal	-	-	-	248.48±1.58^{a}	48.97±1.35^{b}	100.99±0.54^{b}	-	-	-	-	-	-	-	-	-
(E)-2-Pentenal	-	-	-	-	4.49±0.01	-	-	-	-	-	-	-	-	-	-
（E)-2-Hexenal	-	-	-	-	-	-	-	-	-	-	-	-	311.66±8.52^{a}	161.90±1.81^{b}	-
(E)-2-Octenal	-	-	-	-	6.08±0.37^{b}	-	-	-	99.54±3.81^{a}	-	-	-	-	-	-

(E)-2-Nonenal	-	-	-	-	-	-	-	-	-	65.63±8.17^{a}	-	-	-	93.51±2.97^{a}	-
(Z)-2-Decenal	-	-	-	-	-	-	35.88±4.25^{a}	-	-	-	-	-	28.43±0.14^{a}	29.24±0.22^{a}	-
(Z)-4-Decenal	-	-	-	12.83±0.16	-	-	-	-	-	-	-	-	-	-	-
(E)-2-Undecenal	-	-	-	-	-	-	-	-	97.53±0.84	-	-	-	-	-	-
(E)-2-Dodecenal	-	-	-	-	-	-	-	-	-	94.03±10.01^{a}	-	-	-	109.82±3.96^{a}	-
(E, E)-2,4-Heptadienal	-	-	-	-	-	-	-	19.50±0.19	-	-	-	-	-	-	-
2-methyl-Butanal	163.43±6.51^{b}	-	15.63±0.10^{c}	159.19±0.87^{b}	39.80±0.91^{bc}	-	439.04±2.23^{a}	-	-	81.20±5.44^{bc}	17.50±0.24^{c}	-	107.81±1.26^{bc}	-	38.34±0.68^{bc}
3-methyl-Butanal	168.46±8.02^{c}	376.08±0.33^{b}	30.88±0.42^{d}	316.51±2.14^{b}	70.92±4.92^{cd}	68.21±4.40^{cd}	-	40.77±5.77^{d}	-	-	35.70±4.25^{d}	142.45±1.37^{cd}	504.57±4.68^{a}	-	103.57±0.00^{cd}
(E)-2-methyl-2-Butenal	-	-	-	-	9.66±0.00	-	-	-	-	-	-	-	-	-	-

2-ethyl-Butanal	-	-	-	20.90±2.20	-	-	-	-	-	-	-	-	-	-	-
2-ethyl-Hexanal	-	-	-	-	22.13±1.82[a]	-	-	-	-	-	-	-	49.54±3.91[a]	-	-
2-methyl-Undecanal	55.59±1.25[a]	-	-	-	-	-	-	-	-	-	-	-	16.90±1.25[b]	-	-
**Ketones (8)**															
2-Butanone	-	-	-	3871.47±30.97[a]	536.57±29.07[b]	-	-	38.65±5.47[b]	-	-	-	-	255.24±6.73[b]	-	31.09±0.72[b]
2-Octanone	-	-	-	-	-	-	-	-	-	-	-	-	118.88±9.25	-	-
2-Nonanone	-	-	-	-	30.36±1.82[b]	-	-	-	-	-	-	-	382.15±1.53[a]	-	-
2-Undecanone	-	-	2.61±0.25	-	-	-	-	-	-	-	-	-	-	-	-
2-Dodecanone	7.30±0.19[a]	-	-	12.03±1.20[a]	-	-	-	10.99±0.31[a]	-	-	-	-	-	-	-
2-Tridecanone	-	-	-	10.71±0.26	-	-	-	-	-	-	-	-	-	-	-

6-methyl-5-Hepten-2-one	146.07±0.57^{a}	90.55±12.45^{ab}	-	-	-	-	-	-	-	-	110.10±2.22^{ab}	21.30±0.30^{b}	-	-	-
5-methyl-2-Hexanone	15.17±0.14	-	-	-	-	-	-	-	-	-	-	-	-	-	-
**Alcohols (14)**															
1-Pentanol	-	-	-	260.80±2.79^{a}	21.60±0.84^{a}	163.08±1.57^{a}	367.18±3.65^{a}	-	-	-	-	-	-	-	-
1-Hexanol	-	-	-	-	-	226.59±2.25^{a}	267.97±1.25^{a}	-	-	-	-	-	-	133.27±2.09^{a}	-
1-Heptanol	-	-	-	-	-	274.83±8.76^{a}	-	-	-	186.70±7.52^{a}	-	-	-	-	-
2-Octanol	-	-	-	-	0.79±0.00	-	-	-	-	-	-	-	-	-	-
2-Nonanol	13.98±0.06	-	-	-	-	-	-	-	-	-	-	-	-	-	-
2-Undecanol	-	-	-	72.46±7.94^{a}	28.37±1.92^{a}	-	-	-	-	-	17.52±1.10^{a}	-	-	40.84±0.66^{a}	-
1-Penten-3-ol	149.05±2.78^{b}	-	-	305.50±2.68^{b}	82.43±5.44^{b}	-	770.85±2.93^{a}	-	192.19±1.29^{b}	-	-	-	169.92±0.61^{b}	109.55±2.92^{b}	-

3-Penten-1-ol	-	-	-	-	-	-	-	-	-	64.74±2.31	-	-	-	-	-
(Z)-2-Penten-1-ol	55.43±1.90^{b}	-	-	1103.28±9.71^{a}	5.82±0.40^{b}	-	-	-	-	-	85.50±5.30^{b}	47.91±0.00^{b}	90.98±0.44^{b}	-	-
5-Hexen-2-ol	45.20±3.01^{a}	-	-	-	4.02±0.36^{b}	-	-	-	-	-	-	-	-	-	-
1-Octen-3-ol	-	-	-	170.77±1.25^{a}	-	-	70.27±4.84^{ab}	29.68±2.10^{b}	-	-	-	-	-	-	-
(E)-2-Decen-1-ol	25.43±0.19^{a}	-	-	-	-	-	-	-	-	-	4.65±0.55^{b}	-	-	-	-
2-ethyl-1-Hexanol	-	-	-	-	-	-	-	-	-	-	148.47±1.47^{a}	-	-	-	20.21±0.84^{b}
2-methyl-1-Hexadecanol	80.16±0.86^{b}	203.64±6.23^{b}	-	628.97±4.49^{a}	-	-	213.49±1.34^{b}	-	37.97±1.38^{b}	-	52.94±7.35^{b}	-	-	-	-
**Aromatics (12)**															
Benzene	2399.15±98.57^{bcd}	6328.16±42.46^{ab}	92.26±12.43^{d}	9160.08±81.01^{a}	2986.92±18.52b^{cd}	1508.65±91.56^{cd}	4863.09±27.57^{bc}	2444.47±29.91^{bcd}	3919.36±27.21^{bcd}	1890.43±6.29^{cd}	2006.58±10.77^{cd}	2366.88±17.48^{cd}	3481.30±15.08^{bcd}	1839.10±81.13^{cd}	264.21±5.96^{d}

Toluene	801.36±39.36^{c}	1161.33±82.85^{c}	90.43±7.46^{c}	7332.97±6.86^{a}	626.57±23.75^{c}	522.04±2.70^{c}	3991.13±22.85^{b}	243.21±7.93^{c}	430.45±2.97^{c}	372.44±1.86^{c}	453.66±1.14^{c}	557.69±4.60^{c}	918.56±2.34^{c}	690.52±63.47^{c}	72.50±0.81^{c}
Ethylbenzene	72.49±1.52^{c}	-	-	556.15±4.98^{a}	95.40±4.51^{c}	53.74±7.60^{c}	312.49±1.67^{b}	20.38±1.44^{c}	90.05±5.23^{c}	89.71±1.27^{c}	190.04±3.52^{bc}	8.78±0.12^{c}	212.64±2.04^{bc}	109.39±7.02^{bc}	-
p-Xylene	73.85±1.84^{b}	-	-	915.66±8.04a	77.84±3.45^{b}	84.67±11.97^{b}	283.98±9.59^{b}	46.11±3.26^{b}	115.00±8.05^{b}	38.62±2.45^{b}	-	-	279.79±7.29^{b}	112.71±5.74^{b}	-
o-Xylene	-	-	-	455.73±4.16^{ab}	194.45±3.52^{b}	-	632.90±2.06^{a}	-	-	-	-	-	263.49±12.00^{b}	-	-
Styrene	93.66±1.44^{cd}	-	-	352.84±2.81^{a}	117.06±1.46^{cd}	43.54±3.17^{cd}	259.10±9.63^{ab}	-	84.27±1.73^{cd}	33.70±1.14^{d}	179.78±2.00^{bc}	-	169.77±2.21^{bcd}	87.98±6.54^{cd}	-
Propylbenzene	48.22±1.28^{b}	-	-	168.96±1.62^{a}	36.86±2.51^{b}	-	138.26±4.91^{ab}	-	61.60±1.40^{b}	94.58±4.01^{ab}	-	-	-	-	-
Butylbenzene	47.53±0.20^{b}	-	-	147.82±1.26^{a}	25.90±1.70^{b}	55.23±2.20^{b}	-	-	-	96.52±6.05^{ab}	56.10±0.35^{b}	-	-	-	-
Pentylbenzene	34.43±0.51^{bc}	-	-	195.76±1.50^{a}	19.82±0.45^{c}	71.20±4.56^{bc}	-	-	-	111.91±5.32^{ab}	70.13±2.23^{bc}	-	72.43±1.87^{bc}	-	-

Benzaldehyde	331.43±4.57^{cd}	548.59±38.40^{cd}	62.20±0.26^{d}	3717.07±3.01^{a}	882.67±38.62^{bcd}	774.18±5.16^{bcd}	1422.98±5.33^{bc}	132.52±6.60^{d}	770.40±4.04^{bcd}	622.76±2.39^{cd}	262.62±4.36^{cd}	385.43±3.21^{cd}	1909.72±45.99^{b}	1027.57±30.24^{bcd}	279.38±0.94^{cd}
1,2,4-trimethyl-Benzene	-	-	-	211.57±1.63	-	-	-	-	-	-	-	-	-	-	-
Naphthalene	399.30±2.30^{b}	-	-	1177.05±0.54^{a}	-	-	-	-	-	-	245.62±7.57^{b}	204.26±1.20^{b}	426.36±0.70^{b}	-	-
**Furans (3)**															
3-methyl-Furan	-	-	-	-	-	42.78±2.14	-	-	-	-	-	-	-	-	-
Furfural	-	-	-	448.92±3.21^{a}	33.03±2.24^{b}	-	428.16±2.62^{a}	-	-	-	-	-	-	-	-
2-Furanmethanol	261.72±4.53^{b}	-	-	713.79±2.71^{a}	105.41±8.45^{b}	-	684.29±3.68^{a}	-	-	-	-	-	-	-	-
**S-containing compounds (12)**															
Propyl mercaptan	-	-	-	-	-	-	-	-	-	24.08±0.91^{a}	-	19.77±0.28^{a}	-	-	-
1-Nonanethiol	-	-	-	-	-	-	-	-	-	-	-	-	-	369.10±14.54	-

Dimethyl disulfide	-	652.32±65.09^{a}	-	-	126.19±10.73^{a}	-	-	-	-	-	-	-	-	-	-
Dimethyl Sulfoxide	-	-	24.42±0.63^{b}	-	-	-	-	-	-	-	-	269.60±1.21^{a}	-	-	-
Dimethyl trisulfide	-	-	32.15±1.06^{b}	-	123.68±8.89^{ab}	113.02±1.60^{ab}	-	40.31±2.85^{b}	-	278.87±1.80^{a}	-	-	-	-	-
2-methyl-Thiophene	151.93±7.85^{a}	-	-	-	-	-	-	-	-	49.10±3.63^{a}	-	682.95±9.66^{a}	-	-	-
3-ethyl-Thiophene	24.78±1.54	-	-	-	-	-	-	-	-	-	-	-	-	-	-
2-propyl-Thiophene	-	39.12±2.23	-	-	-	-	-	-	-	-	-	-	-	-	-
2-butyl-Thiophene	44.58±0.24^{a}	-	-	-	-	-	-	-	-	58.69±3.16^{a}	33.60±1.99^{a}	-	-	-	-
2,3-dimethyl-Thiophene	20.74±0.44	-	-	-	-	-	-	-	-	-	-	-	-	-	-

Thiazole	-	-	-	-	-	-	-	11.78±0.83^{b}	-	-	-	-	168.22±0.88^{a}	-	-
2-Acetylthiazole	234.53±2.10^{bc}	510.31±44.11^{a}	20.54±1.01^{c}	-	-	-	457.80±2.75^{ab}	108.33±0.98^{c}	-	181.69±7.70^{c}	92.61±0.04^{c}	272.05±1.84^{abc}	-	-	35.57±0.80^{c}
**N-containing compounds (7)**															
Trimethyl amine	1908.87±55.86^{cd}	3632.89±33.14^{ab}	307.25±4.26^{de}	2354.87±18.30^{bc}	159.43±17.91^{e}	186.75±6.97^{e}	1846.32±7.13^{cde}	434.37±1.59^{de}	811.50±2.62^{cde}	1368.15±7.41^{cde}	1108.19±6.55^{cde}	4763.87±21.09^{a}	1349.97±1.88^{cde}	651.59±29.58^{de}	176.54±9.05^{e}
Pyrrole	-	-	-	-	-	-	-	13.05±1.16	-	-	-	-	-	-	-
Pyridine	107.78±8.47^{bcd}	-	-	362.64±3.43^{a}	115.14±4.86^{bcd}	23.94±1.17^{d}	227.93±8.16^{abc}	-	59.38±3.35^{cd}	-	-	32.40±0.46^{d}	259.43±0.11^{ab}	-	-
3-methyl-Pyridine	33.28±1.27^{b}	-	-	388.97±2.89^{a}	-	-	138.24±7.35^{b}	-	-	-	-	-	214.69±5.32^{ab}	-	-
4-methyl-Pyrimidine	68.59±0.33	-	-	-	-	-	-	-	-	-	-	-	-	-	-
3-propyl-Pyridine	-	-	-	-	-	-	210.16±1.92^{a}	-	-	-	-	-	177.36±10.58^{a}	-	-
3-butyl-Pyridine	105.67±2.45^{b}	-	-	268.66±1.57^{a}	-	-	-	-	-	-	-	-	207.42±9.26^{ab}	-	-

**Hydrocarbons (15)**															
Hexane	1537.50±19.28^{bc}	6290.77±38.93^{bc}	85.30±3.63^{c}	18615.82±17.21^{a}	2332.70±112.53^{bc}	751.67±3.43^{bc}	7334.66±22.11^{b}	170.43±1.55^{c}	1465.34±7.91^{bc}	810.33±3.38^{bc}	1772.86±3.67^{bc}	821.24±5.10^{bc}	2810.74±1.95^{bc}	597.31±40.21^{c}	83.67±1.75^{c}
Heptane	226.73±4.55^{b}	-	-	934.82±8.38^{a}	367.81±17.23^{b}	56.05±3.24^{b}	956.35±3.01^{a}	40.12±2.84^{b}	68.45±4.84^{b}	57.70±1.18^{b}	237.50±1.04^{b}	-	300.15±3.41^{b}	42.02±1.51^{b}	-
Octane	221.10±5.02^{cde}	303.13±25.40^{cd}	4.49±0.15^{e}	713.59±6.14^{ab}	441.40±20.66^{bc}	64.66±3.96^{de}	841.61±2.83^{a}	83.84±0.20^{de}	74.31±2.92^{de}	71.69±3.11^{de}	268.00±1.05^{cde}	117.08±0.65^{de}	408.73±4.99^{c}	65.02±0.69^{de}	14.14±0.03^{e}
Nonane	-	-	-	-	674.41±30.76^{a}	-	-	-	-	-	-	-	737.44±6.01^{a}	-	-
Undecane	196.29±4.47^{bc}	278.66±25.90^{bc}	-	907.47±8.31^{a}	-	87.29±5.07^{c}	626.19±3.65^{ab}	-	50.34±1.33^{c}	77.57±5.87^{c}	-	-	265.78±1.35^{bc}	-	-
1-Heptene	39.02±0.80^{cd}	-	-	124.13±5.07^{b}	26.58±2.54^{d}	51.19±3.73^{cd}	177.13±5.99^{a}	-	-	78.16±4.58^{bc}	24.87±1.17^{d}	11.09±0.16^{d}	42.77±1.29^{cd}	24.35±1.13^{d}	-
(Z)-3-Heptene	-	-	-	-	-	7.75±0.34	-	-	-	-	-	-	-	-	-
1-Octene	-	-	-	-	-	20.81±2.05	-	-	-	-	-	-	-	-	-
2-Octene	-	97.88±5.24^{bcd}	-	163.33±1.46^{ab}	34.79±2.50^{cd}	67.16±3.98^{bcd}	195.21±1.16^{a}	22.03±0.17^{d}	-	-	37.49±2.72^{cd}	120.43±0.80^{abc}	22.55±0.29^{d}	45.80±1.29^{cd}	-

1-Nonene	-	-	-	61.22 ±1.62^{a}	-	-	-	-	-	-	-	-	59.61±4.06^{a}	35.40±1.44^{a}	-
cis-4-Nonene	-	-	-	-	-	22.25 ±1.21	-	-	-	-	-	-	-	-	-
1-Decene	-	-	-	-	65.33±4.79^{a}	112.66±6.19^{a}	-	-	-	114.94±7.64^{a}	-	-	-	78.59±2.97^{a}	-
(E)-2-Undecene	-	-	-	-	22.88±1.86^{a}	-	-	-	-	48.41 ±3.99^{a}	-	-	-	-	-
1,3-Pentadiene	-	-	-	-	-	-	268.09±1.17^{a}	35.52±2.47^{b}	-	-	-	-	-	-	-
D-Limonene	-	508.78±8.64^{a}	-	-	104.24 ±8.78^{b}	55.44 ±7.74^{b}	-	-	-	15.85 ±0.61^{b}	450.25±8.07^{a}	-	-	-	-
**Miscellaneous compounds (5)**															
Propanoic acid	-	228.37±9.57^{a}	15.68 ±0.02^{c}	-	-	-	-	-	-	-	122.89±4.56^{b}	-	-	-	11.40 ±1.13^{c}
Nonanoic acid	-	-	-	-	-	-	-	-	-	27.96 ±3.47	-	-	-	-	-
Undecanoic acid	-	-	-	-	-	255.67±6.06^{a}	-	-	-	228.70±9.64^{a}	-	-	-	-	-
Ethyl Acetate	-	-	-	547.21 ±5.20^{a}	28.68±1.04^{b}	-	-	-	-	-	28.31 ±1.92^{b}	-	-	-	-

2-Heptanol, acetate	-	-	-	-	-	-	-	-	-	10.18 ±0.47	-	-	-	-	-

Different letters indicate significant differences according to Analysis of Variance (ANOVA) ($p<0.05$).

"-" indicates that the data was not detected. M=Meat; G=Gonads; H= Hepatopancreas.

SS= Shanghai Baodao Aquaculture Professional Cooperative, Sea & River 21; TS= Taizhou Jiangyuan Animal Husbandry Co., Ltd., Sea & River 21; TC= Taizhou Jiangyuan Animal Husbandry Co., Ltd., Changjiang 2.

Table.S4. Odorant active value (OAV>10) in edible parts of *E. sinensis* from three origins.

Compounds	Female-M			Female-G			Female-H			Male-M			Male-H		
	SS	TS	TC	SS	TS	TC	SS	TS	TC	SS	TS	TC	SS	TS	TC
**Aldehydes (14)**															
Hexanal	-	-	-	-	57.02	19.28	-	59.12	-	14.12	-	32.47	-	11.92	11.73
Heptanal	-	-	-	44.29	13.27	-	36.10	-	-	-	-	-	-	-	-
Octanal	-	-	-	-	-	107.58	-	-	-	-	-	-	-	-	-
Nonanal	87.71	90.35	18.26	247.23	71.82	72.80	141.09	17.31	47.15	104.71	87.78	244.18	-	104.82	18.40
Decanal	-	-	-	2484.80	489.70	1009.90	-	-	-	-	-	-	-	-	-
(E)-2-Hexenal	-	-	-	-	-	-	-	-	-	-	-	-	16.23	-	-
(E)-2-Octenal	-	-	-	-	-	-	-	-	33.18	-	-	-	-	-	-
(E)-2-Nonenal	-	-	-	-	-	-	-	-	-	820.38	-	-	-	1168.88	-
(Z)-2-Decenal	-	-	-	-	-	-	119.60	-	-	-	-	-	94.77	97.47	-
(Z)-4-Decenal	-	-	-	641.50	-	-	-	-	-	-	-	-	-	-	-

(E)-2-Undecenal	-	-	-	-	-	-	-	-	125.04	-	-	-	-	-	-
(E)-2-Dodecenal	-	-	-	-	-	-	-	-	-	67.16	-	-	-	78.44	-
2-methyl-Butanal	163.43	-	15.63	159.19	39.80	-	439.04	-	-	81.20	17.50	-	107.81	-	38.34
3-methyl-Butanal	153.15	341.89	28.07	287.74	64.47	62.01	-	37.06	-	-	32.45	129.50	458.70	-	94.15
**Alcohols (4)**															
1-Hexanol	-	-	-	-	-	40.46	47.85	-	-	-	-	-	-	23.80	-
1-Heptanol	-	-	-	-	-	50.89	-	-	-	34.57	-	-	-	-	-
(Z)-2-Penten-1-ol	-	-	-	12.37	-	-	-	-	-	-	-	-	-	-	-
1-Octen-3-ol	-	-	-	113.85	-	-	46.85	19.79	-	-	-	-	-	-	-
**Aromatics (2)**															
Benzaldehyde	-	13.16	-	89.14	21.17	18.57	34.12	-	18.47	14.93	-	-	45.80	24.64	-
Naphthalene	-	-	-	19.62	-	-	-	-	-	-	-	-	-	-	-
**S-containing compounds (1)**															
2-Acetylthiazole	23.45	51.03	-	-	-	-	45.78	10.83	-	18.17	-	27.21	-	-	-
**N-containing compounds (1)**															
Trimethylamine	795.36	1513.70	128.02	981.20	66.43	77.81	769.30	180.99	338.13	570.06	461.75	1984.95	562.49	271.50	73.56
**Hydrocarbons (3)**															
Hexane	-	-	-	12.41	-	-	-	-	-	-	-	-	-	-	-
1-Octene	-	-	-	-	-	41.62	-	-	-	-	-	-	-	-	-
D-Limonene	-	50.88	-	-	10.42	-	-	-	-	-	-	-	-	-	-
**Miscellaneous compounds (1)**															
Ethyl Acetate	-	-	-	109.44	-	-	-	-	-	-	-	-	-	-	-

"-" indicates that the data was not detected.

M=Meat; G=Gonads; H= Hepatopancreas.

SS= Shanghai Baodao Aquaculture Professional Cooperative, Sea & River 21; TS= Taizhou Jiangyuan Animal Husbandry Co., Ltd., Sea & River 21; TC= Taizhou Jiangyuan Animal Husbandry Co., Ltd., Changjiang 2.
